# Long‐term effects of two 24‐hour moisturizing products on skin barrier structure and function: A biometric and molecular study

**DOI:** 10.1002/hsr2.308

**Published:** 2021-06-06

**Authors:** Aniseh Samadi, Saman Ahmad Nasrollahi, Mahmoud Nateghi Rostami, Zahra Rezagholi, Farideh Abolghasemi, Alireza Firooz

**Affiliations:** ^1^ Center for Research & Training in Skin Diseases & Leprosy Tehran University of Medical Sciences Tehran Iran; ^2^ Pasteur Institute of Iran Tehran Iran; ^3^ Faculty of Pharmacy Tehran University of Medical Sciences Tehran Iran

**Keywords:** 24‐hour moisturizer, aquaporin 3, barrier function

## Abstract

**Introduction:**

Recently, there are a few moisturizers showing hydrating effects up to 24 hours after single application. Aquaporin 3 might be associated with the degree of skin hydration. We aimed to assess the effects of two brands of 24‐hour moisturizers on the skin barrier function, as well as the AQP3 gene expression.

**Method:**

Two moisturizers were applied once daily by 20 participants age 36.15 ± 9.55 years. Upper right and left forearms were randomly assigned to application of each product, whereas the right lower forearm served as control site for application of a cream base formulation. Biophysical assessments including trans epidermal water loss (TEWL), skin hydration, pH, surface lipids, and elasticity parameters were performed before intervention, 1, 4, and 24 hours after single application, following 2 weeks daily application and 1 week after termination of use. Also 5‐mm punch biopsies were performed from application sites of product B and cream base formulation in for five participants after 2 weeks of application.

**Results:**

A single treatment with both products led to 24‐hour increase in skin moisture in comparison with the control site (*P*‐value <.01). Daily application of both products for 14 days also led to significant improvement in skin moisture (*P*‐value <.01), TEWL (*P*‐value <.01), and elasticity parameters. The increase in skin hydration was associated with upregulation of AQP3 gene expression in treated area for one of the formulations (*P*‐value = .04).

**Conclusion:**

The tested 24‐hour moisturizers only need to be applied once daily to improve skin barrier function and hydration and up‐regulate AQP3 mRNA expression.

## 1 INTRODUCTION

Water is vital for the natural function and appearance of healthy skin. Skin hydration is the result of the cooperation of three main mechanisms: stratum corneum and its barrier role for water loss; natural moisturizing factor (NMF), including several hygroscopic molecules to maintain hydration in the corneocytes, and water‐transporting channels.^1^

Water‐transporting proteins, aquaporins (AQPs), have the key role in providing proper skin hydration and maintaining water balance in the cell layers.^2^ Up to four different AQPs (AQP1, 3, 9, 10) are selectively expressed in human skin epidermis; however, aquaporin 3 (AQP3) is the most abundant one. It is an aquaglyceroporin, facilitating osmotically driven water transport across the cell plasma membrane.^3^ The activity level of AQP3 in the epidermis showed to be associated with the degree of skin hydration through ex‐vivo studies.^4^, ^5^

Moisturizers are major components of daily skin care, commonly used to improve skin hydration.^6^ Recently, a few studies have shown moisturizing effects of a single application of some formulations on the skin up to 24 hours.^7^, ^8^ Cosmetic ingredients used to increase skin hydration act through different mechanisms, such as providing occlusive conditions, attracting and holding water molecules on the skin, and enhancing the skin's own hydrating properties to stimulate moisture production.^9^

AQP3 is a biomarker of skin hydration and could be a key target for moisturizing effects of cosmetic ingredients via intracellular pathways. Here, it is hypothesized that long‐term skin hydration of 24‐hour moisturizers might be explained by increased expression of AQP3. To the authors' knowledge, there is no in‐vivo study to show such an association in 24‐hour products. Thus, the purpose of this study is to assess the effects of two types of 24‐hour moisturizers on the skin barrier function and AQP3 gene expression.

## 2 MATERIAL AND METHODS

### 2.1 Study design and participants

It was a single‐center, randomized, controlled, intrasubject, double‐blinded, 3‐week study. Twenty healthy participants (19 females and 1 male) with a mean age of 36.15 ± 9.55 years and a clinical diagnosis of dry skin were enrolled after signing their written informed consent. Seven participants had skin type III and 13 had type IV. Participants were excluded in case of a positive history of major skin diseases or current smoking, as well as undergoing systemic corticosteroid or cytostatic therapy within the past 2 weeks. They were also excluded in case of using topical drugs that might influence skin hydration within 7 days. Other exclusion criteria were the presence of any condition on the inner forearms interfering with a skin assessment and pregnancy or breastfeeding.

The study was performed in compliance with the Declaration of Helsinki, and the study protocol was approved by the ethics committee of the National Institute for Medical Research Development in Iran (Acceptance code: IR.NIMAD.REC.1397.405). It also was registered in the Iranian Registry of Clinical Trials with an approved code of IRCT20190210042676N4 before conduction. All participants signed informed written consent for participation in the study.

### 2.2 Test preparations

Two moisturizer creams available in the Iran market were used in current study, as described in detail in Table 1. A simple hydrophilic cream base was used as the control formulation.

**TABLE 1:** Moisturizing products used in this study

Product	Ingredients	pH
A	Aqua, glycerin, caprilic/capric triglyceride, shea butter, *Imperata cylindrica* extract, coconut oil, emulsifier, *Macadamia nut oil, Mangifera indica* seed oil, squalene, tocopherol acetate, bisabolol, borage oil, Q10	6.32
B	Aqua, glyceryl glucoside, *Simmondsia chinensis* seed oil, hydrogenated coco‐glycerides, caprylic‐capric‐triglyceride, isopropyl palmitate, synthetic beeswax, glyceryl stearate citrate, distarch phosphate, octyldodecanol, pentylene glycol, panthenol or dexpanthenol, cetyl alcohol, stearyl alcohol, biotin (vitamin B7), glycine, alanine, carbomer, tocopheryl acetate, dimethicone, caprylyl glycol, phenoxyethanol	5.28
Cream base	Aqua, cetyl alcohol, stearic acid, propylene glycol, and propyl paraben	6.25

### 2.3 Study protocol

Participants underwent a conditioning period of 3 days prior to the study. No application of topical products to the inner forearms was allowed during this period to ensure that there are no residual effects from product application. Participants were also instructed not to wash the forearms within 3 hours of arrival at the test facility. Upperparts of right and left forearms randomly were assigned to the daily application of products A and B, while the right lower forearm was considered as the control site for the daily application of the cream base formulation.

### 2.4 Biometric assessment

Skin biophysical parameters, including TEWL (g/m^2^/h), SC hydration (arbitrary units), elasticity (arbitrary units), erythema index (arbitrary units), skin surface lipid index (μg/cm^2^), and skin pH were measured before the intervention, as well as 1, 4, and 24 hours after a *single application* of creams, after 2 weeks daily application and 1 week after stopping the application. In the 2‐week application phase, the final measurements were done 24 hours after the last application. All measurements were performed using respective probes, TEWAmeter, Corneometer, Cutometer, Sebumeter, and pHmeter (Courage & Khazaka electronic GmbH, Cologne, Germany) by the same investigator as previously reported by the authors.^10^

Before biometric measurement, the participants rested in a room with climate control, having a temperature of 22°C ± 2°C and relative humidity of 30% to 40% for 30 minutes. The same investigator performed the same measurements in each participant.

### 2.5 Skin biopsy, isolation of total RNA, and real‐time PCR

Due to logistic and financial limitations, AQP3 gene expression was measured only in sites of application of product B and cream base formulation (as control site) in five participants.

At the end of 2 weeks application, a 5‐mm punch biopsy specimen was obtained from application sites of product B and cream base formulation. Skin samples were kept frozen in RNAlater stabilization solution (Invitrogen, Termo Fisher) to be analyzed for AQP3 mRNA expression levels.

Standard precautions were employed to avoid RNase contamination and RNA degradation. Tissues were crushed into a very fine powder using a mortar and pestle system under liquid nitrogen.

After homogenization, for each sample, 1 mL of AccuZolTM (Bioneer, Daejeon, South Korea) was added, followed by the addition of 0.2 mL of chloroform (Merck, Darmstadt, Germany) and the centrifugation for 15 minutes at 12 000*g* at 4°C. RNA was precipitated from the aqueous phase by adding 0.5 mL of isopropanol (Merck) and washed with 70% ethanol (Merck). Total RNA content was then analyzed by measuring 260/280 nm ratios of absorbance values via BioTek PowerWave HT Scanning Microplate Spectrophotometer (BioTek, Winooski, Vermont).

Reverse transcription of isolated RNA was performed using the cDNA synthesis kit, using the manufacturer's instruction (Biothchrabbits GMbH, Berlin, Germany). The sequences of primers used for real‐time RT‐PCR are shown in Table 2. Quantification analysis of the Syber green RT‐PCR was carried out using MIC PCR software version 2.4.0 (Applied Biosystems, Foster City, California).

**TABLE 2:** Sequence of primer pairs for AQP3 and β‐actin

Name	Bac‐F 5′➔3′	Tm	Bac‐R 5′➔3′	Tm
β‐Actin	CTGGCACCCAGCACAATG	59	CATCTGCTGGAAGGTGGACA	59.7
Name	Aqp‐F 5′➔3′		Aqp‐R 5′➔3′	
AQP3	GTGAGCCCTGGATCAAGCTG	60.7	TTGGGGCCCGAAACAAAAAG	59.5

For real‐time RT‐PCR, 25 μL reaction mixtures were prepared, using 12.5 μL Ampliqon real plus 2x master mix green (Odense M, Denmark), 3 μL cDNA, 2 μL primer pair mix (0.5 mM each) and 7.5 μL dH_2_O.

The levels of AQP3 mRNA expression were normalized to the respective levels of the housekeeping gene β‐actin. Relative fold expression was calculated using threshold‐cycle (Ct) and following formula:

2^−ΔΔCt

### 2.6 Randomization and blinding

It was a simple randomization. The areas assigned to the test products A and B were randomized in each participant using a random number table.

Both preparation and cream base formulation were packaged in similar anonymous jars, distinguished with different codes. The application site of each product was added on the label according to the random list by the third independent person. The investigator, who performed the assessment, was also blind to the application site of each product.

### 2.7 Statistical analysis

Sample size calculation was performed with a hypothesis to find a positive difference in skin hydration between treated and control sites 24 hours after a single treatment of at least 20%. With an effect size of 0.6, an alpha value of 0.05, and a power of 80%, a total of at least 20 participants should be enrolled to detect this difference between the active treated sites vs the control.

The SPSS software version 18 (SPSS, Inc, Chicago) was used for statistical analysis. The repeated measure ANOVA test was used for the analysis of the study outcomes in order to compare treated sites vs the control at each time point, as well as skin hydration and TEWL in several follow‐up visits vs baseline. The statistical significance level was defined as *P* < .05.

## 3 RESULTS

Twenty participants (1 man and 19 women) with a mean age of 36.15 years (SD: 9.55) were enrolled in the study and all completed it.

The baseline data for each parameter were compared among two test sites and the control site using ANOVA with repeated measurement tests, showing no significant difference for none of the evaluated parameters. P‐values for baseline measurements were 0.27, 0.76, 0.65, 0.78, 0.71, and 0.79 for skin hydration, TEWL, pH, R2, R5, and skin sebum, respectively.

### 3.1 Skin hydration

**FIGURE 1:**
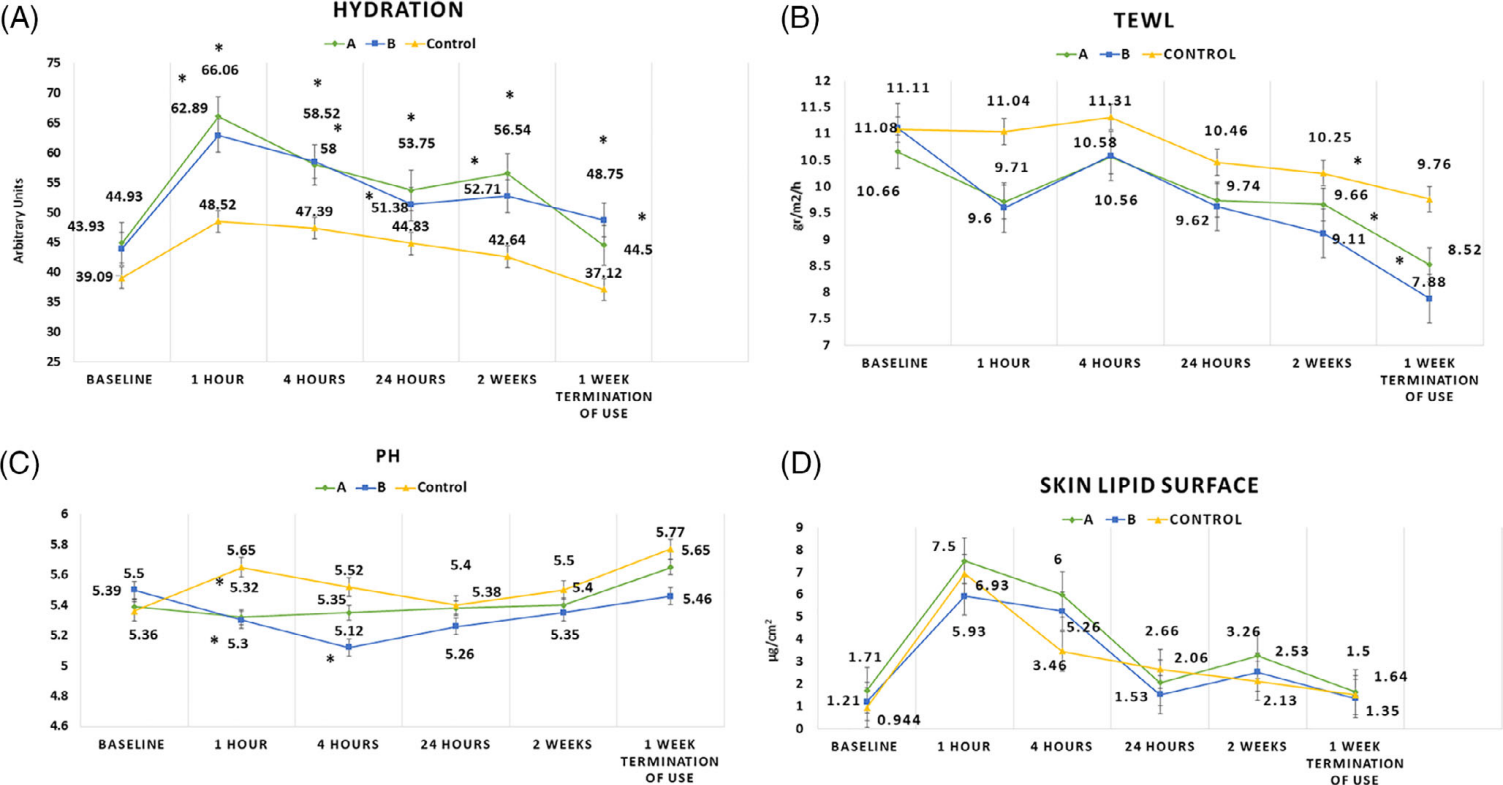
(A‐D) Skin hydration, trans epidermal water loss, skin pH, and skin lipid surface for products A and B as well as cream base control site, 1, 4, and 24 hours after single application as well as after 2 weeks repeated application (*significant compared to control site, *P* < .05)

1, 4, and 24 hours after a single application and after 2 weeks daily application, both products A and B showed significantly higher skin hydration compared with the control site (*P*‐value <.01). In none of the time points, a significant difference was detected between products A and B (Figure 1A).

One week after stopping the application, skin hydration level remained significantly higher in application sites of both products compared to the control site (*P*‐value <.01 and .03 for products A and B, respectively).

In control sites, no statistical modification in skin hydration was observed at each time point evaluation in comparison with baseline.

### 3.2 Trans epidermal water loss

After a 2‐week application of product B, a significant decrease was detected in TEWL, which remained significant even 1 week after termination of use (*P*‐value <.01; Figure 1B).

In the case of product A, a significant decrease occurred in TEWL after 2 weeks of application compared to the control site; however, the decrease was not significant after stopping the application.

### 3.3 Skin pH

Skin pH reduced significantly 1 hour after the application of both products and in the application site of product B, the decrease remained significant until 4 hours (*P*‐value <.01). An ANOVA with repeated measurement showed no significant differences between products A and B (Figure 1C).

### 3.4 Skin lipid surface

No significant difference was detected in skin surface lipid content in none of measurement time points for none of preparations compared to the control (Figure 1D).

### 3.5 Skin elasticity

**FIGURE 2:**
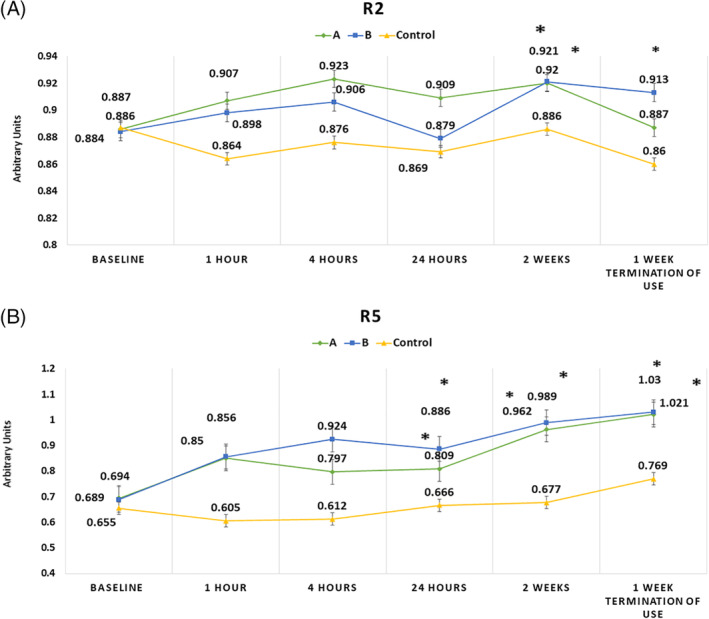
Skin elasticity parameters; R2 (A) and R5 (B) for products A and B as well as cream base control site, 1, 4, and 24 hours after single application as well as after 2 weeks repeated application (*significant compared to control site, *P* < .05)

Gross elasticity (R2) significantly increased after 2 weeks application of both products compared to the control site (*P*‐value .043 and .035, respectively); however, this improvement continued till 1 week after termination of product B (*P*‐value = .05; Figure 2A).

Improvement in net elasticity (R5) compared to the control site was observed 24 hours after the first application, the 2‐week application, as well as 1 week after termination of both products (*P*‐value <.01; Figure 2B).

No significant difference was detected in the skin erythema index after the application of tested products compared to the control site in measurement times.

### 3.6 Real‐time PCR for AQP3 mRNA expression

The relative quantities of the target genes were normalized against internal standard gene (β‐actin). Threshold cycles (Cts) of amplified templates of test were used for calculation of different gene expressions using 2̂‐ Δ Δ Ct method.

The mean level of AQP3 expression in the site treated with product B was compared with the control site, and relative fold expression was reported. Results showed that the upregulation of AQP3 gene expression in areas treated with product B was 4.84 times higher than the control site (cream base treatment), and this difference is statistically significant (*P* = .048; Table 3).

**TABLE 3:** Relative expression of AQP3 genes in treated site using product B comparing control site (day 14)

	Mean ± SD	*P*‐value
Δ Ct for treated site using product B	4.76 **±** 2.84	.048
Δ Ct for control site	7.21 **±** 3.69	
mRNA expression in treated area using product B /control site (2̂‐ Δ Ct)	4.84 Range (0.18‐16.56)	

## 4 DISCUSSION

This report was the first in‐vivo study with a molecular assessment on 24‐hour moisturizers. The findings showed that a single treatment with either product A or B led to a 24‐hour enhancement of the skin's moisture. Daily application of both products for 14 days also led to significant improvement in skin moisture, TEWL, and elasticity parameters compared with the vehicle‐controlled area, indicating that the moisturizers might only need to be applied once daily.

Comparing two product, product B showed a superior improving effect on TEWL and elasticity parameters after the termination of use. It also led to significant upregulation of AQP3 gene expression compared to the control.

Two products contain different active ingredients. Glyceryl glucoside (GG) is the active ingredient of product B, which is a derivative of the endogenous glycerol composed of a natural combination of glycerol and glucose. Glycerol has proper interaction with the water‐binding and hydrophilic properties of SC.^11^, ^12^ Schrader et al. have shown that GG is effective in reducing TEWL and increasing the AQP3 mRNA levels.^13^ The mechanism of the upregulation of AQP3 with GG has not been completely explained. However, GG could actively be transported to keratinocytes in dehydrating circumstances, altering the extreme osmotic environment via AQP3 biosynthesis.^14^

To the authors' knowledge, the present work is the first molecular assessment on 24‐hour moisturizers; the increased level of AQP3 could explain the improving trend of TEWL and skin hydration even after stopping the product application. Additionally, in an in‐vivo setting, we confirmed the ex vivo report of Caverzan et al, showing that reduced TEWL and promoted human skin hydration are related to modulation of the AQP3.^15^ Stimulating the biosynthesis of aquaporin is also associated with an increasing level of CD44, claudin‐1, and filaggrin proteins involved in skin water maintenance.^16^

*Imperata cylindrical* is the active ingredients of product A, which has hydrating characteristics due to the presence of natural osmoprotective compounds, including 3‐dimethylsulfoniopropionate, potassium, and sugars, in the formulation.^17^, ^18^

Another active ingredient in product A is lauric acid, which is found naturally in coconut oil and could influence skin moisture via producing eicosanoid, increasing membrane fluidity, and cell signaling.^19^, ^20^

A further consideration is the decreasing effect of product B on skin pH compared to the control site, which is not detected in product A. AQP3 is a functional pH‐sensitive water channel^3^; lower skin pH induced by product B could be another triggering factor for the AQP3 expression.

The results show a significant improvement in skin elasticity parameters (R2 and R5) after the application of both moisturizers, which in the case of product B, continues 1 week after stopping the application.

R2 (gross elasticity) measures the total skin stretch, including viscous deformation, which represents the perceived stretchiness of hydrated skin. R5 (net elasticity) is independent of viscous deformation; it is used to characterize the mechanism of skin movement, testing specifically the tensioned nature of the skin.^21^

Improving skin elasticity is primarily described by increasing the water content of SC.^22^ Moreover, product B contains hyaluronic acid (HA), which is an essential part of the extracellular matrix of basal keratinocytes. HA interferes with keratinocyte proliferation and migration,^23^, ^24^ facilitates the rearrangement of dermal collagen fibers, and leads to advanced elasticity properties.^25^

The antiaging effects of coenzyme Q10 and shea butter (containing a high percentage of triterpenes, tocopherol, phenols, and sterols) should also be considered.^26^, ^27^

The current study had some limitations, the most important of which was the small number of participants, yet the results showed significant differences. In the case of molecular assessment, only five participants were biopsied, which probably would not be enough to conclude but confirm the clinical observations. Due to logistic and financial limitations, only the product with better moisturizing effect (according to previous reports) was selected for molecular assessment. Further studies, with AQP3 assessment after application of the other product and measurement of additional markers of interest could make a more comprehensive understanding of the results.

## 5 CONCLUSION

In conclusion, the present study demonstrated the long‐term moisturizing effect of two 24‐hour products after a single application. As the first molecular study on 24‐hour moisturizers, the upregulation of AQP3 gene expression was also explained as a possible molecular pathway for their long‐term moisturizing properties.

## CONFLICT OF INTEREST

The authors declare there is no conflict of interest.

## AUTHOR CONTRIBUTIONS

Conceptualization: Alireza Firooz, Saman Ahmad Nasrollahi

Data curation: Aniseh Samadi, Zahra Rezagholi. Farideh Abolghasemi

Formal analysis: Aniseh Samadi

Investigation: Aniseh Samadi, Zahra Rezagholi. Farideh Abolghasemi

Methodology: Alireza Firooz, Saman Ahmad Nasrollahi, Mahmoud Nateghi Rostami

Project administration: Alireza Firooz

Writing—original draft preparation: Aniseh Samadi

Writing—review and editing: Saman Ahmad Nasrollahi, Mahmoud Nateghi Rostami, Alireza Firooz

  Aniseh Samadi conducted the biometric part, performed the statistical analysis, and also wrote the initial draft; Zahra Rezagholi and Farideh Abolghasem contributed in research and data acquisition. Mahmoud Nateghi Rostami conducted the molecular part. Saman Ahmad Nasrollahi revised the manuscript. Basic concept and idea were conceived by Alireza Firooz.

  All authors critically revised the initial draft and final manuscript.

## TRANSPARENCY STATEMENT

The lead author affirms that this manuscript is an honest, accurate, and transparent account of the study being reported; that no important aspects of the study have been omitted; and that any discrepancies from the study as planned (and, if relevant, registered) have been explained.

## ETHICS STATEMENT

This study was approved by the ethics committee of National Institute for Medical Research Development in Iran (Acceptance code: IR.NIMAD.REC.1397.405). It is also registered in Iranian Register of Clinical Trials before conduction with approved code of IRCT20190210042676N4. Data were generated, recorded, and processed in accordance with the Declaration of Helsinki and principles of Good Clinical Practice. The method of study was explained to all volunteers, and a written informed consent was obtained.

## Data Availability

Data sharing is not applicable to this article due to ethical and commercial restrictions.
